# Society Meetings

**Published:** 1902-06

**Authors:** 


### SOCIETY MEETINGS.

THE American Surgical Association will hold its 20th annual meeting at Albany, June 4, 5 and 6, 1902.

THE March meeting of the Physicians' League of Buffalo was postponed and held at the home of Dr. Cora B. Lattin on April 7. Dr. Ida C. Bender, read a paper on the necessity of sanitary inspection in the public schools, and Dr. Helene Kuhlman read a paper on Dementia precox.

THE 11th annual meeting of the Association of Military Surgeons of the United States will convene in Washington, D.C., on Thursday morning, June 5, 1902, and continue in session during the two following days. Every member is cordially urged to be present and participate in all the exercises, both social and literary.

THE Medical Society of the County of Erie will hold its 81st semiannual meeting in the parlors of the Y.M.C.A. building, Tuesday, June 17, 1902, at 10 o'clock a.m., under the presidency of Dr. W. M. Ward, of North Collins. The following program has been arranged: Nasal deformities and their correction, John O. Roe, Rochester; The state license to practise medicine, William Warren Potter, Buffalo, John M. Lee, Rochester, and Lee H. Smith, Buffalo, presidents of the State Medical examining boards; discussion opened by Dr. Henry R. Hopkins, president of the Medical Society of the State of New York. Congenital pulmonary stenosis, Joseph Burke, Buffalo. Presentation of a case of aortic aneurism, William C. Krauss, Buffalo.

THE United States Pension Examiners of the State of New York will hold a meeting at Saratoga Springs, June 9, 1902. The following program has been prepared: Address of welcome, E. Valencourt Deuell, president of the Saratoga Board; Defects in reports of examining surgeons, with suggestions for their improvement, J. F. Raub, medical referee Pension Bureau, Washington, D.C.; discussion to be opened by William Warren Potter, Buffalo. The influence and value of organisation, George W. Miles, Oneida; discussion to be opened by W. L. Ayer, Owego. The advantages of organisation from the point of view of the special examiner, Wheelock Rider, Rochester; discussion to be opened by John N. Oliver, Brooklyn. All pension examining surgeons in the state are cordially invited to attend. Dr. William A. Howe, of Phelps, is the secretary, who may be addressed for further particulars.

---

THE Buffalo Academy of Medicine held meetings during the month of May as follows:

*Section on Surgery.*—Tuesday evening, May 6. Program: An experimental and clinical research into surgical shock and collapse, illustrated by stereopticon, Geo. W. Crile, Cleveland, O.

*Section on Medicine.*—Tuesday evening, May 13. Program: Jaundice, with report of interesting cases, J. M. Anders, Philadelphia; A case of acromegaly, F. S. Crego; Heredity, Wm. C. Krauss.

*Stated Meeting.*—Tuesday evening, May 20. Program furnished by Section on Pathology: Dysentery and its causes, Simon Flexner, Professor of Pathology, University of Pennsylvania; Notes on a few cases of smallpox from the Buffalo epidemic of 1901-1902 (stereopticon), David E. Wheeler.

*Section on Ophthalmology, Otology, Laryngology and Rhinology.*—Monday evening, May 26. Program: All members of the Academy interested in this section are requested to be present to decide if meetings shall be continued. Annual election of officers of this section.

---

THE Erie County Medical Association held a meeting in Buffalo, at the University Club, 295 Delaware Ave., Thursday, May 15, 1902, at 8.30 p.m. The program was: Colles's fracture, John Parmenter; leaders of discussion: W. C. Phelps, Eugene A. Smith. Brewers' yeast in therapeutics, Julius Ullman; leader of discussion: Charles G. Stockton.

The officers are: President, Joseph W. Grosvenor, Buffalo; vice-president, Howard L. Hunt, Orchard Park; secretary, Jacob

S. Otto, Buffalo; treasurer, William J. Thornton, Buffalo; members of executive committee, A. A. Hubbell, Bernard Cohen, Lorenzo Burrows, Jr., all of Buffalo.

---

THE American Orthopedic Association will hold its 16th annual meeting at Philadelphia, June 5, 6, and 7, 1902.

---

THE Rochester Academy of Medicine, section on surgery, held its regular monthly meeting at the Genesee Valley Club, Wednesday, May 14, 1902, at 8.15 p.m. The following program was observed: The management of middle-ear suppuration, Wendell C. Phillips, New York (by invitation); discussion opened by F. Whitehill Hinkel, Buffalo (by invitation), Thomas H. Halsted, Syracuse (by invitation), John O. Roe and Wheelock Rider. Edward W. Mulligan, chairman. William M. Brown, secretary.

---

THE National Confederation of State Medical Examining and Licensing Boards will hold its twelfth annual meeting in the hall of the Y. M. C. A., Saratoga Springs, N. Y., Monday, June 9, 1902.

